# Physiological and Biomechanical Determinants of Sprint Ability Following Variable Intensity Exercise When Roller Ski Skating

**DOI:** 10.3389/fphys.2021.638499

**Published:** 2021-03-25

**Authors:** Trine M. Seeberg, Jan Kocbach, Jørgen Danielsen, Dionne A. Noordhof, Knut Skovereng, Pål Haugnes, Johannes Tjønnås, Øyvind Sandbakk

**Affiliations:** ^1^Centre for Elite Sports Research, Department of Neuromedicine and Movement Science, Norwegian University of Science and Technology, Trondheim, Norway; ^2^Smart Sensor Systems, SINTEF Digital, Oslo, Norway; ^3^Mathematic and Cybernetics, SINTEF Digital, Oslo, Norway

**Keywords:** cross-country skiing, gross efficiency, skiing technique, maximal oxygen consumption, inertial measurement unit, Near-infra red spectroscopy, physiological determinants, biomechanical determinants

## Abstract

The most common race format in cross-country (XC) skiing is the mass-start event, which is under-explored in the scientific literature. To explore factors important for XC skiing mass-starts, the main purpose of this study was to investigate physiological and biomechanical determinants of sprint ability following variable intensity exercise when roller ski skating. Thirteen elite male XC skiers performed a simulated mass-start competition while roller ski skating on a treadmill. The protocol consisted of an initial 21-min bout with a varying track profile, designed as a competition track with preset inclines and speeds, directly followed by an all-out sprint (AOS) with gradually increased speed to rank their performance. The initial part was projected to simulate the “stay-in-the-group” condition during a mass-start, while the AOS was designed to assess the residual physiological capacities required to perform well during the final part of a mass-start race. Cardiorespiratory variables, kinematics and pole forces were measured continuously, and the cycles were automatically detected and classified into skating sub-techniques through a machine learning model. Better performance ranking was associated with higher VO_2*Max*_ (*r* = 0.68) and gross efficiency (*r* = 0.70) measured on separate days, as well as the ability to ski on a lower relative intensity [i.e., %HR_*Max*_ (*r* = 0.87), %VO_2*Max*_ (*r* = 0.89), and rating of perceived exertion (*r* = 0.73)] during the initial 21-min of the simulated mass-start (all *p*-values < 0.05). Accordingly, the ability to increase HR (*r* = 0.76) and VO_2_ (*r* = 0.72), beyond the corresponding values achieved during the initial 21-min, in the AOS correlated positively with performance (both *p* < 0.05). In addition, greater utilization of the G3 sub-technique in the steepest uphill (*r* = 0.69, *p* < 0.05), as well as a trend for longer cycle lengths (CLs) during the AOS (*r* = 0.52, *p* = 0.07), were associated with performance. In conclusion, VO_2*Max*_ and gross efficiency were the most significant performance-determining variables of simulated mass-start performance, enabling lower relative intensity and less accumulation of fatigue before entering the final AOS. Subsequently, better performance ranking was associated with more utilization of the demanding G3 sub-technique in the steepest uphill, and physiological reserves allowing better-performing skiers to utilize a larger portion of their aerobic potential and achieve longer CLs and higher speed during the AOS.

## Introduction

Cross-country (XC) skiing is a physiologically and technically demanding endurance sport where speed, work rate, and energy expenditure fluctuate with the constantly changing terrain (Andersson et al., 2010, 2016; Sandbakk et al., 2011; Sandbakk and Holmberg, 2014; Bolger et al., 2015). The variation between relatively short sections of uphill, flat and downhill terrain challenge XC skiers to alternate between different sub-techniques with varying contributions from leg and arm work within the two main styles, skating and classic (Seeberg et al., 2017; Solli et al., 2018; Tjønnås et al., 2019).

Accordingly, successful XC skiing requires a high maximal oxygen uptake (VO_2*Max*_), as well as the ability to reach a high peak oxygen uptake (VO_2*Peak*_) and to ski efficiently within the different sub-techniques (Sandbakk and Holmberg, 2017). Since XC skiers generate particularly high work rates on uphill terrain (Sandbakk et al., 2012a; Andersson et al., 2016; Sandbakk and Holmberg, 2017; Haugnes et al., 2019a), pushing the metabolic demands considerably above those required to elicit VO_2*Max*_, XC skiing additionally requires sufficient levels of anaerobic capacity and the ability to recover and reproduce anaerobic power during competitions (Losnegard et al., 2012; Gløersen et al., 2018, 2020; Karlsson et al., 2018; Losnegard, 2019).

Efficient skiing in such constantly changing terrain requires frequent shifts between the different sub-techniques and inherent regulation of cycle length (CL) and cycle rate (CR; Pellegrini et al., 2013; Solli et al., 2018). Previous research has shown that faster skiers use more demanding sub-techniques in steeper terrain than slower skiers (Andersson et al., 2010; Marsland et al., 2017). Additionally, while more efficient skiers obtain longer CL (Sandbakk et al., 2010, 2012a,b, 2013; Åsan Grasaas et al., 2014), fast skiing also requires the ability to employ rapid cycles when accelerating at the start, during breakaway attempts and when sprinting at the finish of races (Haugnes et al., 2019b). In this context the understanding of how skiers regulate the power contributions from poles and skis to generate the required propulsion, and how this affects the oxygenation of muscles in arms and legs is unclear, especially in the skating technique.

The influence of the above-mentioned performance-determining variables on performance in XC skiing could differ between race formats. For example, sprint skiers have different physiological characteristics, with higher muscle mass and anaerobic power, than performance-matched distance skiers who are able to produce a higher aerobic power (Losnegard and Hallén, 2014). However, the most common race format in XC skiing, the mass-start events, are virtually unexplored (Losnegard, 2019), and the impact of physiological and biomechanical performance-determining variables for mass-start performance is currently unknown.

Mass-start competitions are performed on the same race-tracks as time trials. However, since many skiers are racing together, the tactical elements will play a greater role for the result and could also influence the physiological and biomechanical demands. Mass-starts are commonly decided by a mass sprint or by a sprint between a few remaining contestants, and more seldom by a single skier crossing the finish solo after a breakaway. In all cases, high capacity to produce aerobic and anerobic power, together with high efficiency in the most important sub-techniques, should enable skiers to work at a lower relative intensity to follow the pace, and thereby reduce the accumulation of fatigue before entering the final sprint.

The extent to which physiological and biomechanical variables determine the different components and the overall performance in mass-start XC skiing competitions may be valuable information to further optimize training and competition strategies. To explore factors important for XC skiing mass-starts, the main purpose of this study was to investigate physiological and biomechanical determinants of sprint ability following variable intensity exercise when roller ski skating.

## Materials and Methods

### Overall Design

**FIGURE 1:**
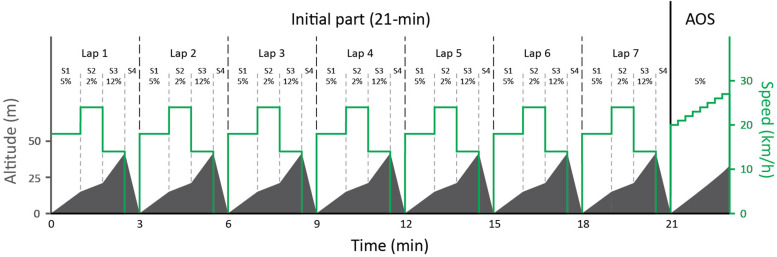
Protocol showing the changes in altitude and speed for the simulated mass-start competition divided into a 21-min initial part with seven 3-min laps where each contains 4 segments (S), followed by an all-out incremental sprint (AOS) with gradually increased speed each 15 s until exhaustion. Although the track was designed for the use of specific sub-techniques in each segment, the skiers could freely select sub-techniques themselves during the entire test.

In this study, we measured physiological and biomechanical variables in elite skiers performing a simulated mass-start [i.e., variable intensity exercise followed by an all-out sprint (AOS)], while roller ski skating on a treadmill. The track was organized as seven identical 3-min laps consisting of four different segments simulating a moderate uphill (S1), a flat segment (S2), a steep uphill (S3), and a simulated downhill (S4; Figure 1). The profile of the track was designed according to standards of the International Ski Federation, where the following sub-techniques could naturally be utilized (Andersson et al., 2010): gear 2 (G2), a technique for skiing uphill that involves an asymmetrical double pole push in connection with every other leg push; gear 3 (G3), a technique used on moderate inclines and level terrain that involves one double pole push together with every leg push; gear 4 (G4), a symmetrical double pole push in connection with every other leg push, used on level terrain; and gear 7 (G7), when the skier is in a downhill deep stance position without moving poles or legs. Although the track was designed for the use of specific sub-techniques in each segment, the skiers could freely select sub-techniques themselves.

The protocol consisted of two consecutive parts with the same preset load for all skiers: (1) a low-intensity familiarization during a 21-min bout on the simulated competition track and (2) the simulated mass-start competition, where the initial 21-min part on the simulated competition track was performed at high intensity and immediately followed by an AOS, with gradually increasing speed until exhaustion. The initial 21-min part was projected to simulate the “stay-in-the-group” condition of a mass-start, while the AOS was designed to assess the residual physiological capacities required to perform well during the final part of a mass-start race. Oxygen uptake (VO_2_), heart rate (HR), near infrared spectrometry (NIRS), kinematics and pole forces were monitored continuously, while blood lactate concentration (BLa), and rating of perceived exertion (RPE) were measured directly after each part. In addition, performance-determining variables [gross efficiency (GE) and VO_2*Max*_] were measured on a separate day.

### Participants

Thirteen elite male Norwegian skiers, consisting of eight XC skiers (distance FIS points: 47 ± 21) and five biathletes, participated in the study (Table 1). All skiers were healthy and free of injuries at the time of testing. The skiers were instructed to prepare in the same manner as before a competition, but with no strenuous exercise the last 24 h before the test. All skiers were conversant with treadmill roller skiing and VO_2_ measurements from previous testing sessions and daily training routines.

**TABLE 1:** Anthropometric, physiological, and performance characteristics [mean value ± standard deviation (SD)] of the thirteen male skiers participating in the study.

Variables	Mean value	*SD*
Age (years)	24.8	*2.7*
Body height (cm)	184	*6.0*
Body mass (kg)	79.3	*5.2*
Body mass index (kg⋅m^–2^)	23.4	*1.0*
VO_2*Max*_ (mL⋅min^–1^⋅kg^–1^)	69.5	*3.6*
VO_2*Max*_ (mL⋅min^–1^)	5505	*364*
HR_*Max*_ (beat⋅min^–1^)	193.5	*7.0*
Skinfold thickness triceps brachii arm (mm)	6.6	*1.8*
Skinfold thickness vastus lateralis leg (mm)	7.3	*1.3*

*HR_*Max*_: The highest measured heart rate (beat⋅min^–1^). VO_2Max_: The highest 30-s moving average (based on 10-s mixing chamber values) during the incremental maximum test in the pretest.*

### Equipment

#### Laboratory and Ski Equipment

The protocol was performed on a 3-by-5-m motor-driven treadmill on roller ski (Forcelink S-mill, Motekforce Link, Amsterdam, Netherlands). The skiers used poles of their individually chosen lengths with special carbide tips. All skiers wore their own skating XC shoes but used the same pair of skate elite roller skis (IDT Sports, Lena, Norway) with an NNN binding system (Rottefella, Klokkarstua, Norway) and with standard category 2 wheels to minimize variations in roller resistance. The rolling friction coefficient (μ) was tested before, at various times during, and after the study using the towing test described by Sandbakk et al. (2010), providing an average μ-value of 0.016.

The visual movement of the skiers were captured from behind with a video camera (GoPro Hero6, Inc, San Mateo, CA, United States). The skiers wore a safety harness connected to an automatic emergency brake at the high intensity parts of the tests. Incline and speed of the treadmill were calibrated before and after the study by using the Qualisys Pro Reflex system and Qualisys Track Manager software (Qualisys AB, Gothenburg, Sweden).

#### Physiological Measurements

Before testing, the body mass of each skier was determined with an electronic body-mass scale (Seca model nr:877; Seca GmbH & Co. KG., Hamburg, Germany). Respiratory variables were measured continuously using open-circuit indirect calorimetry (Oxycon Pro, Erich Jaeger GmbH, Hoechberg, Germany). Expired gas was passed through a mixing chamber and analyzed continuously. The instruments were calibrated against ambient air and a commercial gas with known concentrations of O_2_ (15%) and CO_2_ (5.85%) before the start of each test. The flow transducer (TripleV, Erich Jaeger GmbH, Hoechberg, Germany) was calibrated using a 3-L high-precision calibration syringe (5530 series, Hans Rudolph Inc., Kansas City, MO, United States). The data were collected as 10-s mixing chamber values and are given as body weight adjusted oxygen uptake (VO_2_) and as percentage of VO_2*Max*_ (%VO_2*Max*_).

Garmin Forerunner 920XT (Garmin Ltd., Olathe, United States) was used to continuously measure HR at a sampling frequency of 1 Hz. Relative HR (%HR_*Max*_) was calculated as % of maximal HR (HR_*Max*_) for each skier, and HR_*Max*_ was defined as the highest measured value for each person measured at any time during the tests. BLa was measured using Biosen C-line Sport lactate measurement system (EKF Industrial Electronics, Magdeburg, Germany) collecting 20 μL blood form the fingertip. The device was calibrated every 60 min with a 12-mmol μL standard concentration. RPE for upper body, lower body and overall were recorded using the 6–20-point Borg Scale (Borg, 1982).

#### NIRS Measurements

Muscle oxygenation was assessed using a wireless NIRS system (Portamon, Artinis Medical Systems, Netherlands) consisting of two optodes, each with three transmitters and one receiver. All transmitters emitted light at wavelengths of 760 and 850 nm and used a sample rate of 10 Hz. The optode sites were shaved before placement. The two optodes were placed on the vastus lateralis of the right leg and the long head of the triceps brachii on the right arm and secured with tape and elastic bandages before they were covered with a black cloth to prevent the interference of ambient light. At the end of the test, skinfold thickness was measured (three times) at the sites of optode placement using a skinfold caliper (Holtain skinfold caliper, Holtain Ltd, Crymych, United Kingdom), see Table 1. The data from the different NIRS sensors was collected and synchronized in time by the designated software and the tissue saturation index (TSI) with a Fit factor higher than 99.8% was used in the study. In order to remove the resulting 1-s gaps in the NIRS-data, it was chosen to interpolate with the average value of the two neighbor points. Here TSI_*leg*_ is TSI from the sensor placed on vastus lateralis of the right leg, and TSI_*arm*_ is TSI from the sensor placed on the long head of the triceps brachii on the right arm.

#### Movement and Pole Force Data

Eight Oqus 400 infrared cameras captured 3D position of passive reflective markers placed bilaterally on the body, on roller skis and poles with a sampling frequency of 200 Hz. The specific body locations of the reflective markers were on the ski boot at the distal end of the fifth metacarpal, the lateral malleolus (ankle), lateral epicondyle (knee), greater trochanter (hip), lateral end of the acromion process (shoulder), lateral epicondyle of humerus (elbow), and styloid process of ulna (wrist). One marker was placed on the lateral side of each pole, ∼5 cm below the handle, and one marker was placed on the lateral side of the pole tips, for calculation of pole direction and thus direction of pole forces. For ski measurements one marker was placed 1 cm behind the front wheel, and one marker 1 cm in front of the back wheel of each roller ski. The motion capture system only measured every second lap (lap 1, 3, 5, and 7) during the simulated mass-start, to reduce risk for overload of data and system failure.

Instrumented ski pole grips (Proskida, Whitehorse, YT, Canada) were used to measure the axial (resultant) force directed along the poles. The data was streamed to a mobile phone via the Bluetooth protocol, and later downloaded to a computer and synchronized with the movement data. The sampling frequency of the force data was 100 Hz.

An IMU placed on the front of the chest (Physiolog 5 from GaitUp SA, Lausanne, Switzerland) was used to provide continuous motion data for automatic detection and classification of the skating sub-techniques and the corresponding movement pattern. The IMU consisted of a 3D-accelerometer and 3D-gyroscope with sampling frequency 256 Hz in addition to a barometric pressure sensor with sampling frequency 64 Hz. Data was stored locally on the sensor during the test and later downloaded to a computer.

### Protocol

The preparation consisted of attaching the wearable sensors to the body, then the skiers sat passively for 5 min to create a data basis for the NIRS measurements before standing still on the treadmill for 4 min to get a baseline for the respiratory measurements. The active protocol started with a short calibration procedure for the IMU sensor before the 18-min warm up was performed at low to moderate intensity [5 min of G3 at 10 km⋅h^–1^and 5% incline before two 4-min stages using G2 and G4 (10 km⋅h^–1^ at 8% incline)] as part of the NIRS calibration.

The 21-min low-intensity familiarization in the competition track was performed on the treadmill following the pre-set terrain profile (see Figure 1) and set speeds (S1:14 km/h, S2: 20 km/h, and S3: 8 km/h). Thereafter, a 5-min recovery period was given before the initial 21-min part of the simulated mass-start protocol, simulating the “stay-in-the-group” condition, was performed on the same inclines, but at higher speeds (S1:18 km/h, S2: 24 km/h, and S3: 14 km/h). The bout was immediately followed by an incremental AOS to determine sprint abilities required during the final part of a mass-start race. The AOS was performed at 5% incline starting at 20 km⋅h^–1^ and with a 1 km⋅h^–1^ increase in speed every 15 s (see Figure 1). Each skier could freely choose sub-technique and received continuous visual and verbal feedback concerning the upcoming terrain and the time till the next segment but was blinded to the performance of the other skiers.

Based on pilot testing and on performance level of the participants, speeds were chosen so that some skiers (the less good ones) would likely not manage to complete the whole 21-min protocol (simulating that the skiers were not able to “stay-in-the-group”), while some skiers (the best ones) would be well able to complete it. Accordingly, at any time during the mass-start competition, skiers could take an unlimited number of 30-s breaks (by grabbing the rope at the front of the treadmill, simulating tuck). The protocol would keep running regardless, and so the skier would continue skiing wherever after each such 30-s break. The skiers were clearly instructed about this opportunity before the start and explained that such a break would simulate a real-life competition situation in which they felt they could no longer keep up with the front, and after a 30-s break they would be skiing together with another group of trailing skiers. They were blinded to the results by the other skiers and were told to still aim for a best possible time in the AOS and thereby achieving the highest rank possible in the mass-start. The overall performance ranking used in the statistics was determined from time-to-exhaustion during the AOS in addition to the number of breaks, where all those finishing the initial 21-min protocol without breaks were ranked before those requiring one, two or three breaks, respectively.

### Protocol for Measuring Performance-Determining Variables

To obtain performance-determining variables while roller ski skating (e.g., VO_2*Max*_ and GE), additional laboratory measurements were conducted on a separate day within 1 week prior to the simulated mass-start. This protocol consisted of a 5-min standardized low-intensity warm-up on the treadmill before each skier performed a total of twelve 4-min bouts with set speed/incline at four different intensity levels, starting with the lowest level. For every intensity level, the three different bouts with specified skiing techniques were performed in randomized order (G2: 12% incline at 6/7/8/9 km⋅h^–1^, G3: 5% incline at 10/12/14/16 km⋅h^–1^, and G4: 2% incline at 15/18/21/24 km⋅h^–1^), and approximately 2 min recovery was given between each stage. The corresponding speeds for each technique were chosen to obtain similar RPE and BLa across sub-techniques for each intensity level. The inclines employed represent typical inclines where these techniques are employed by elite skiers and were based on previous research (Pellegrini et al., 2013; Stöggl and Holmberg, 2016). After the last submaximal exercise bout, a 15 min recovery period (rest and easy warm up) was followed by a maximal incremental test. The starting incline and speed were 10.5% and 11 km⋅h^–1^, after which the speed was kept constant, while the incline was subsequently increased by 1.5% every minute until 14.0%. Thereafter, the speed was increased by 1 km⋅h^–1^ every minute until exhaustion. VO_2_ was monitored continuously and the highest 30-s moving average (based on 10 s mixing chamber values) was defined as VO_2*Max*_.

The submaximal data from this protocol was used to calculate GE as the external work rate divided by the metabolic rate, in accordance with Sandbakk et al. (2010). The metabolic rate was calculated from the average VO_2_ of the last min of each submaximal exercise bout and the oxygen equivalent, using the associated average respiratory exchange ratio and standard conversion tables (Massicotte et al., 1996). The work rate was calculated as the sum of power against gravity [*Pg* = *m* ⋅ *g* ⋅ sin (α) ⋅ *v*] and friction [*Pf* = *m* ⋅ *g* ⋅ cos (α) ⋅μ⋅ *v*]; where *m* is the mass of the skier, *g* the gravitational acceleration 9.81 m/s^2^, α the angle of treadmill incline, *v* the treadmill speed and μ the frictional coefficient. In this paper the average GE was calculated (based on 11 or 12 submaximal exercise bouts; one subject did not complete the G2 exercise bout at the highest intensity, because of a BLa above 4 mmol⋅L^–1^ at the previous intensity) and used in addition to mean values of each sub-technique, i.e., G2, G3, and G4.

### Data Analysis

#### Cycle Detection and Classification of Sub-Techniques

The accelerometer data from the IMU (placed on the chest) was used to automatically detect and classify each individual cycle into a sub-technique using Gaussian filtering and a trained support vector machine learning model with a similar method as used in Rindal et al. (2018). Subsequently, the data was manually examined and corrected for errors in classification by comparing the classified cycles with the video and the graphic representation of filtered accelerometer signals. The accuracy of the model on these data was above 99%. The cycle detection was based on the sidewise movement of the upper body, with cycle start defined at the point when the upper body is in a left position and with the lowest acceleration. Cycle detection together with the treadmill speed were used to derive CL and CR of each cycle. The cycles were classified into the sub-techniques G2, G3, G4, or Other, where Other included G5, transitions between sub-techniques, simulated downhill (G7) and not-skiing activities. The algorithms for cycle detection, model development and classification of sub-techniques were implemented in Matlab R2018b from MathWorks.

#### Calculation of Power-Distribution Between Poling and Ski Push-Offs

The marker position data and pole force data were low pass filtered (8th order Butterworth, 15 Hz cut-off) before further procession. Force and kinematics were synchronized offline (in MATLAB) for each lap recorded by detecting the first instance of pole touch down on the treadmill belt. This touchdown was defined as the first instant when the pole force reached 10 N. The body center of mass (CoM) was calculated based on the position data and body segments mass properties according to de Leva (1996). CoM velocity was obtained by numerical differentiation of position data. In skate style XC skiing, power is generated either by the poles or the skis. Instantaneous pole power (*P*_*Pole*_) was calculated from pole force (*F*_*Pole*_) and CoM velocity (*V*_*CoM*_): [*P*_*Pole*_ = *F*_*Pole*_*__*x*_*⋅*V*_*CoM*_*__*x*_* + *F*_*pole*_*__*y*_*⋅*V*_*CoM*_*__*y*_* + *F*_*Pole*_*__*z*_*⋅*V*_*CoM*_*__*z*_*] with *x*, *y*, and *z* representing components of *F*_*Pole*_ and *V*_*CoM*_ in the forward-backward (*x*), sideways (*y*), and vertical (*z*) directions (Donelan et al., 2002). *P*_*Pole*_ was calculated independently for each pole first, and then summed. The difference between work rate (*P*_*Cycle*_) and cycle average *P*_*Pole*_ was interpreted as average ski power (*P*_*Ski*_). Relative *P*_*Pole*_ (%*P*_*Pole*_) and relative *P*_*Ski*_ (%*P*_*Ski*_) was calculated as % of *P*_*Cycle*_ for each skier, and relative *P*_*PoleLeft*_/*P*_*PoleRigth*_ (%*P*_*PoleLeft*_/%*P*_*PoleRight*_) was calculated as % of *P*_*Pole*_ for each skier. Two of the skiers had missing power data for the whole test due to technical issues and are therefore not included in the power calculations.

#### Synchronization of Data and Definitions

All sensor data (HR, VO_2_, TSI_*Leg*_, TSI_*Arm*_, CL, CR, sub-technique, *P*_*Cycle*_, %*P*_*Pole*_, %*P*_*Ski*_, %*P*_*PoleLeft*_, and %*P*_*PoleRight*_) were synchronized in time to a common master timeline and compound into one dataset with 1 Hz resolution before the means were calculated. Time offsets from the master timeline for treadmill speed and incline, HR, VO_2_, and NIRS data were manually recorded during the data collection. Time offsets for IMU-derived data (CL, CR, and sub-technique) were found based on identifying three synchronization jumps in the IMU data and on video. Reduction to 1-Hz resolution was done by calculating the mean for each second of data, which was the case for all types of data except the NIRS data where the mean 1-Hz values were calculated over three seconds to remove 1-s gaps resulting from the abovementioned filtering.

**FIGURE 2:**
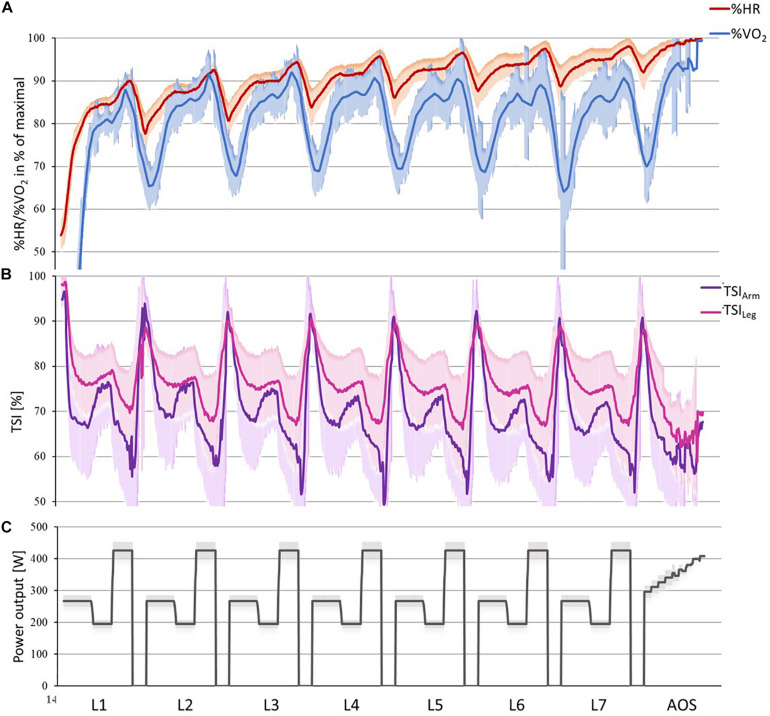
Mean values and standard deviation with 1-Hz resolution for **(A)** heart rate (HR) and oxygen uptake (VO_2_; 30-s moving average) and **(B)** tissue saturation index for the vastus lateralis of the right leg (TSILeg) and the long head of the triceps brachii on the right arm (TSIArm), and **(C)** power output in the simulated mass-start for the 9 skiers who were able to finish the entire protocol without requiring breaks. The data for the 4 skiers who needed breaks followed the same pattern.

**FIGURE 3:**
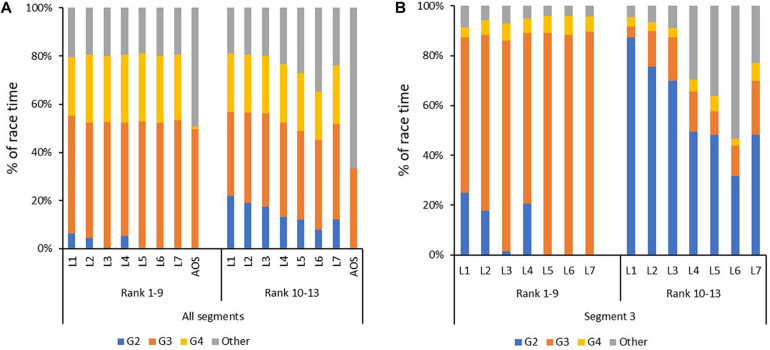
Distribution of sub-techniques [in % of time] for each of the seven 3-min laps (L) during the simulated mass-start, with the skiers divided into two groups: **(A)** for all four segments in the initial 21-min and the all-out sprint (AOS; see Figure 1 for the protocol) and **(B)** for the steepest uphill. Skiers ranked 1–9 are those who were able to finish the entire protocol without requiring breaks and rank 10–13 consists of the 4 lowest ranked skiers that required one or more breaks to complete protocol.

**FIGURE 4:**
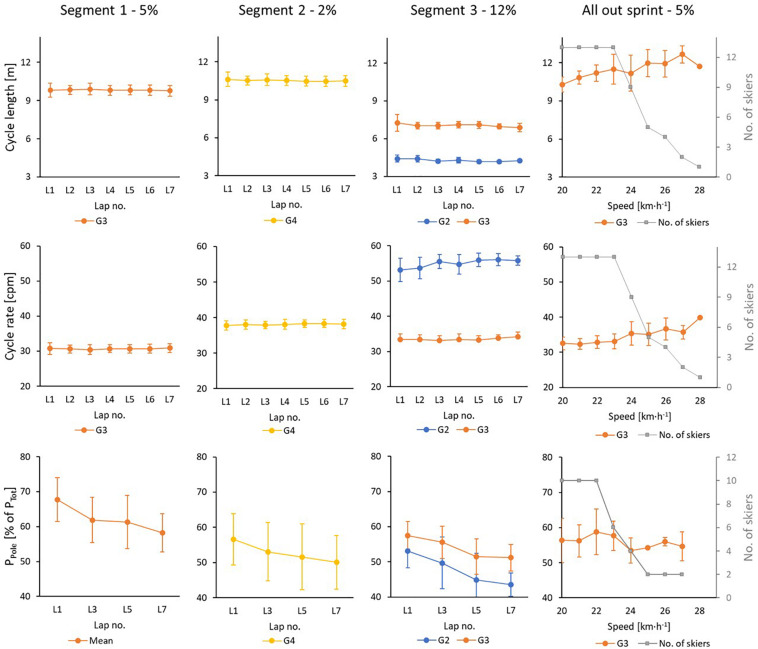
Cycle length, cycle rate [in cycles per minute (cpm)] and body-mass normalized poling power (in %-age of mean cycle power) for the 13 skiers during a 21-min initial part with seven 3-min laps (L) where each contains 4 segments (with three of these analyzed here), followed by an all-out incremental sprint (AOS) with gradually increased speed each 15 s until exhaustion. In AOS the skiers are gradually stopping due to exhaustion, the number of remaining skiers is showed in gray color on the right axis.

When comparing mean values for the skiers according to performance, the period from lap 1 to lap 3 in the mass-start were used instead of all laps, to be able to include all skiers. This was also the case when comparing mean values for the mass-start AOS, here only the first three steps were used. When comparing use of sub-techniques in Figure 3, the skiers were divided into two groups according to their performance-ranking, group 1 consisted of the nine skiers that completed the protocol as planned, and group 2 consisted of the remaining four skiers that needed one or more 30-s breaks due to exhaustion. Difference between segments and drift for physiological values (VO_2_, HR, and TSI) were calculated only for group 1 due to the (slightly) different load for group 2 in the last 4 laps, while drift in kinetic and kinematic variables (power, CL, and CR) were calculated for all skiers since this was linked to a specific sub-technique and thereby the breaks were automatically excluded (classified as Other). For the same reason Figure 2 shows only data for group 1, while Figure 4 shows data for all skiers.

### Statistical Analysis

All variables are presented as mean values for each skier in Tables 2, 3. Before calculating lap-to-lap drift and differences between segments, all data were tested for normality using a Shapiro–Wilk test in combination with visual inspection of data. One-way ANOVA with Tukey’s HSD *post hoc* test was used for analyzing differences in the measured physiological variables between the segments, with the first lap being excluded from the analysis. A paired sample *t*-test was used to examine lap-to-lap drift, with drift in physiological variables being defined as the difference in mean values of lap 7 min lap 2 (to compensate for the delayed kinetic response on lap 1 due to starting from rest). Drift in kinetic and kinematic variables (power, CL, and CR) was defined and calculated as the difference in mean values of lap 7 min lap 1.

**TABLE 2:** Physiological responses measured during the simulated mass-start (MS) and performance-determining variables measured during a separate day for the 13 individual skiers involved in this study.

Performance			MS – mean values (Lap 1 – Lap 3)					MS – after		MS – peak values				Performance determining variables				
				
Rank	30 s Breaks	TTE [s]	HR [% of HR_*Max*_]	VO_2_ [% of VO_2*Max*_]	VO_2_ [mmol⋅ L^–1^⋅ kg^–1^]	TSI_*Arm*_ [%]	TSI_*Leg*_ [%]	RPE [1–20]	Bla [mmol L^–1^])	PeakHR [bpm]		PeakVO_2_ [mL⋅min^–1^⋅kg^–1^]		VO_2*Max*_ [mL⋅min^–^^1^⋅kg^–1^]	Gross efficiency [%]			
	#	AOS	IP	IP	IP	IP	IP	IP	AOS	IP	AOS	IP	AOS		G2	G3	G4	OA
1		130	83.5	74.2	54.7	59.4	61.3	16	10.5	182	193	70.5	74.3	73.8	17.7	15.9	12.7	15.4
2		119	85.1	73.2	53.4	43.8	51.9	17	7.2	184	190	71.7	73.4	73.0	17.4	15.1	12.6	15.0
3		101	87.3	78.2	53.2	45.2	55.3	16	14.3	171	177	69.4	71.0	68.1	17.0	14.9	12.0	14.7
4		91	90.2	74.9	55.0	45.0	58.1	18	12.0	199	202	73.1	71.7	73.4	17.3	15.4	13.0	15.2
5		82	88.0	73.2	53.0	58.5	64.6	15	8.7	187	189	67.6	67.7	72.5	16.9	14.8	12.9	14.9
6		74	88.8	76.7	56.8	36.0	51.4	17	12.9	203	202	72.7	71.4	74.0	16.4	14.5	12.1	14.4
7		65	88.5	79.3	51.3	47.8	57.3	20	10.2	191	189	66.1	61.8	64.8	17.4	14.9	12.8	15.0
8		60	90.6	78.0	53.4	50.1	63.3	19	12.0	193	194	66.1	65.0	68.5	17.3	15.0	12.9	15.1
9		47	89.0	79.4	53.2	65.3	69.7	17	11.8	193	189	68.6	62.9	67.0	17.1	15.1	12.7	15.0
10	1	50	93.7	81.6	58.8	54.4	65.2	20	16.7	202	200	72.6	69.1	72.0	15.8	14.0	11.7	13.8
11	2	62	92.6	83.8	54.7	42.4	46.5	19	18.7	195	196	69.3	63.3	65.3	16.7	14.6	12.1	14.3
12	2	47	91.5	86.6	55.2	63.8	55.6	19	15.1	201	196	67.3	61.1	63.8	16.5	14.5	12.0	14.4
13	3	66	91.1	82.9	56.2	51.0	54.1	20	12.0	189	190	70.9	70.6	67.8	16.2	14.0	12.1	14.1

*Values collected during the simulated mass-start are shown either for the initial part (IP) or after the all-out sprint (AOS). Rank, ranking in the simulated mass-start; TTE, time to exhaustion; HR, heart rate; HR_*Max*_, the highest measured heart rate; VO_2_, oxygen uptake; VO_2Max_, The highest 30-s moving average (based on 10 s mixing chamber values) during the incremental maximum test; TSI_*Leg*_, tissue saturation index for the vastus lateralis of the right leg; TSI_*Arm*_, tissue saturation index for the long head of the triceps brachii on the right arm; RPE, rate of perceived exhaustion; BLa, blood lactate concentration; and Peak HR/VO_2_, the highest measured HR/VO_2_ during the specified bout.*

**TABLE 3:** Distribution of sub-technique, power and cycle characteristics (mean values) measured during the initial part (IP; lap 1 to lap 3) of the simulated mass-start or during the first three steps of the all-out sprint (AOS).

	Sub-technique distribution				Power distribution					Cycle characteristics	
			
	G2 [%]	G3 [%]	G4 [%]	Other [%]	%*P*_*Cycle*_ [Watt]	%*P*_*Pole*_ [% of *P*_*Cycle*_]	%*P*_*Ski*_ [% of *P*_*Cycle*_]	%*P*_*PoleLeft*_ [% of *P*_*Cycle*_]	%*P*_*PoleRigth*_ [% of *P*_*Cycle*_]	CL [m]	CR [cpm]

Rank	IP	IP	IP	IP	IP	IP	IP	IP	IP	AOS	AOS
1	0	55	23	22	283.3	60.9	39.1	48.3	51.6	10.9	32.2
2	4	53	24	19	292.5	52.1	47.9	43.9	56.1	11.5	30.6
3	12	42	24	22	ND	ND	ND	ND	ND	10.8	32.4
4	4	53	23	20	270.5	60.5	39.5	46.6	53.4	10.6	33.0
5	0	57	24	20	305.2	58.6	41.4	43.3	56.6	12.0	29.3
6	8	47	25	19	261.0	58.1	41.9	51.5	48.5	11.0	31.8
7	4	51	24	20	332.9	69.7	30.3	51.1	48.9	10.6	33.1
8	1	56	24	18	288.1	60.4	39.6	48.1	51.9	10.1	34.7
9	0	32	48	20	281.7	60.1	39.9	52.4	47.1	11.2	31.6
10	23	34	25	17	267.0	47.8	52.2	50.5	49.4	10.7	32.8
11	12	45	23	20	ND	ND	ND	ND	ND	10.0	35.2
12	21	36	23	21	286.3	59.8	40.2	44.8	55.2	10.9	32.3
13	22	33	26	19	300.2	50.7	49.3	46.6	53.4	10.1	35.0

*Rank, ranking in the simulated mass-start; ND, no data; %*P*_*Cycle*_, mean cycle power; %*P*_*Ski*_, power from ski push offs in % of *P*_*Cycle*_; %*P*_*Pole*_, power from poling in % of *P*_*Cycle*_; %*P*_*PoleLeft*_, Power from left pole in % of *P*_*Pole*_; %*P*_*PoleRigth*_, Power from right pole in % of *P*_*Pole*;_ CL, cycle length; and CR, cycle rate.*

Correlations between performance ranking [determined from time to exhaustion (TTE) in the AOS, with those requiring one or more breaks during the mass-start being placed behind those with less breaks, independent of TTE] and the different variables, were calculated using the Spearman’s product-moment correlation coefficient. In addition, Pearson’s correlation coefficient between TTE and the different variables for the 9 skiers that completed the entire protocol without breaks (group 1) was calculated. The interpretation of the magnitude of linear association between the variables were evaluated according to Hopkins et al. (2009) as trivial: *r* < 0.1, small: 0.1 ≤ *r* < 0.3, moderate: 0.3 ≤ *r* < 0.5, large: 0.50 ≤ *r* < 0.7, very large: 0.7 ≤ *r* < 0.9, and extremely large: 0.9 ≤ *r* < 1. The level of statistical significance was set at α = 0.05, and 0.05 < α < 0.10 was regarded as trends. All statistical analyses were performed using IBM SPSS Software Version 26.0 (SPSS Inc., Chicago, IL, United States).

## Results

Individual mean values for physiological capacities from the simulated mass-start and performance determining variables are provided in Table 2 and kinematic variables and power distributions are provided in Table 3. The dynamics of the physiological variables during the simulated mass-start for the nine skiers that completed the entire protocol without breaks (group 1) are displayed in Figure 2, the skiers that needed breaks in order to complete (group 2) are not included due to a different load. Mean values of each sub-technique as a function of lap number for both groups are being shown in Figure 3, and CL, CR and power distribution as a function of lap number and sub-technique for each segment for all skiers are given in Figure 4.

All physiological variables fluctuated according to simulated terrain, although a delay in the physiological measured response was present (Figure 2). %HR_*Max*_ in the moderate uphill (S1) was significantly lower than %HR_*Max*_ in the preceding downhill segment [−4.1 ± 1.4 percentage points (pp), *p* = 0.02] and the steep uphill (S3; −5.4 ± 1.4 pp, *p* = 0.002), while it did not differ significantly from %HR_*Max*_ in the flat segment (S2). %VO_2*Max*_ in the moderate uphill (S1) was significant lower than %VO_2*Max*_ in all other segments, with it being 8.0 ± 2.0 pp lower than during the preceding downhill (S4; *p* < 0.001), −14.3 ± 2.0 pp lower than during the flat segment (S2; *p* < 0.001) and −12.1 ± 2.0 pp lower than during the steep uphill (S3; *p* = 0.001). In addition, TSI_*Leg*_ and TSI_*Arm*_ also fluctuated according to the specified terrain segments (Figure 2), but there were no significant differences between the mean values for the different segments. There was a significant lap-to-lap drift in HR (7.9 pp, *p* < 0.001), in TSI_*Arm*_ (−3.0 pp, *p* = 0.007) and in power distribution between poling and ski push off’s (−7.7 pp, *p* = 0.006). However, no significant drift in TSI_*Leg*_, VO_2_, CL, or CR was present.

**FIGURE 5:**
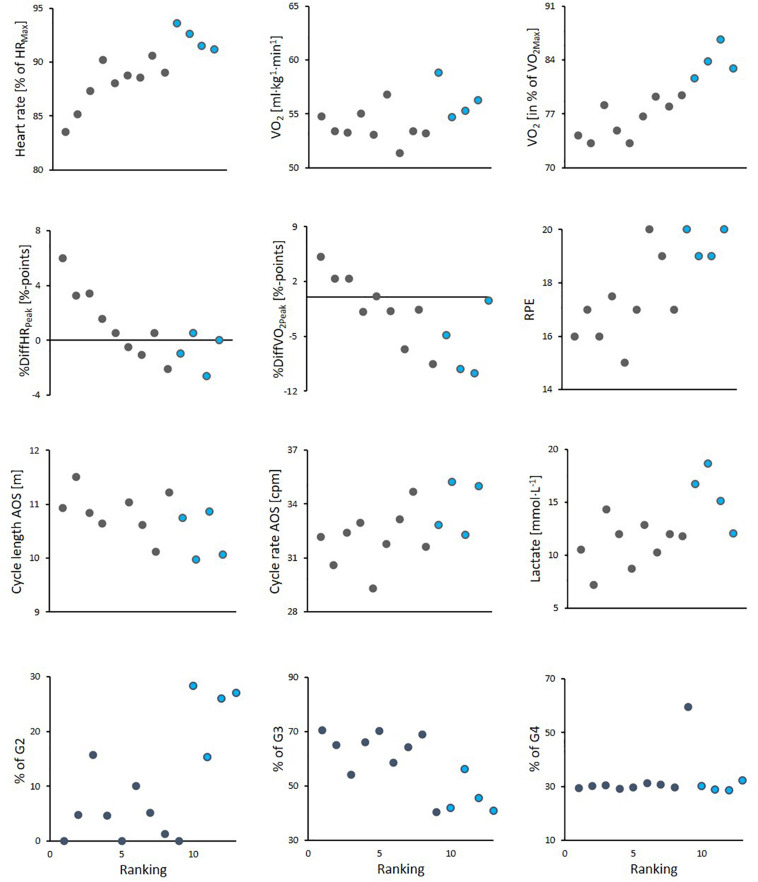
Scatter plots for athlete ranking in the simulated mass-start with the following parameters: mean values of heart rate (HR), oxygen uptake (VO_2_) and sub-technique selection during the first three laps of the simulated mass-start, as well as to the rate of perceived exhaustion (RPE) for the initial 21-min of the mass-start, cycle length and cycle rate during the first 3 steps of the incremental all-out sprint (AOS), blood lactate concentration (BLa) measured directly after the simulated mass-start and the difference between peak values of relative heart rate (%HR_*Max*_) and oxygen uptake (%VO_2Max_) in the main part of the mass-start and the corresponding values during the AOS (%DiffHR_*Peak*_/%DiffVO_2Peak_). The 9 skiers completing the entire protocol without breaks are shown with black color, while the 4 skiers who needed breaks are shown in blue color.

**FIGURE 6:**
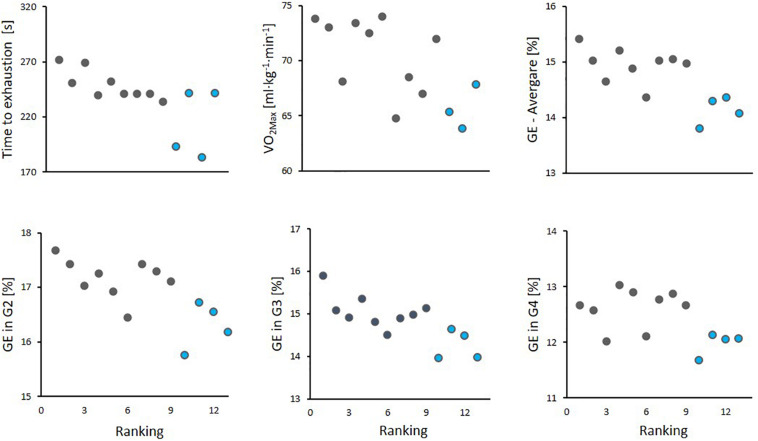
Scatter plots for athlete ranking in the simulated mass-start with the following performance-determining variables: time to exhaustion in the maximal performance test, maximal oxygen uptake (VO_2Max_) and gross efficiency (GE) (average for all sub-techniques (average), and for G2, G3 and G4 sub-techniques) for all 13 skiers. The 9 skiers completing the entire protocol without breaks are shown with black color, while the 4 skiers who needed breaks are shown in blue color.

An overview of significant correlations between variables measured during the simulated mass-start and the performance-determining variables are presented in Table 4 and Figures 5, 6. Here, %HR_*Max*_ and %VO_2*Max*_ during the simulated mass-start showed large- to extremely large correlations with performance, while the body-mass normalized VO_2_ and VO_2*Max*_ (Figure 6) displayed large correlations with performance. Accordingly, the ability to increase HR and VO_2_ in the AOS, beyond the corresponding values achieved during the initial 21-min, showed very large correlation with mass-start performance (Figure 5). In addition, RPE during the mass-start showed a very large correlation and BLa measured directly after the AOS a large correlation with performance. Sub-technique selection (distribution of G2 vs G3) during the main part of the mass-start showed a large correlation with performance, with the best-performing skiers using more G3 (Figure 3). Average GE and specific GE in G2 and G3 had very large correlation with performance, while GE obtained using G4 correlated only moderately with performance. A longer CL and a lower CR in G3 during the AOS was largely correlated with performance. There was considerable subject-to-subject variation in power distribution from poles and skis, and between power produced by the left and right pole, but no significant correlations to performance (Figure 4).

**TABLE 4:** Spearman’s rank order correlation (*R*_*S*_; for all 13 skiers) and Pearson’s correlation (*R*_*P*_; for the 9 skiers who completed the entire protocol without breaks) between ranking (Spearman’s), respectively, time to exhaustion (Pearson’s) in the simulated mass-start (MS) and variables measured during the MS in addition to performance determining physiological variables (PDV) measured on a separate day.

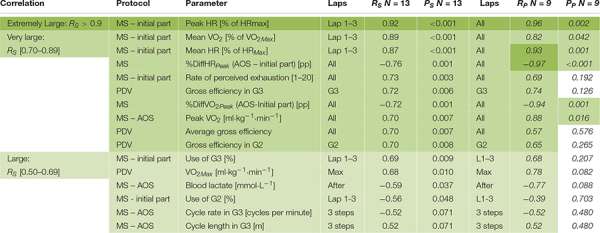

*The MS is divided into the initial 21-min part and the all-out sprint (AOS). HR, heart rate; HR_*Max*_, the highest measured heart rate; HR_*Max*_, the highest value measured during all tests; VO_2_, oxygen uptake; Peak HR/VO_2_, the highest HR/VO_2_ measured during the specified bout; VO_2Max_, the highest 30-s moving average measured (based on 10-s mixing chamber values) during the incremental maximum test; *R*s, Spearman’s rank order correlation coefficient; *P*_*S*_, *p*-value for Spearman’s rank order correlation;%DiffVO_2Peak_, the difference between peak values of relative oxygen uptake (%VO_2Max_) in the main part of the mass-start and during the AOS; %DiffHR_*Peak*_, the difference between peak values of relative heart rate (%HR_*Max*_) in the main part of the mass-start and during the AOS; pp, percentage points; *R*_*P*_, Pearson’s correlation coefficient; and *P*_*P*_, *p*-value for Pearson’s correlation coefficients.*

## Discussion

The primary purpose of this study was to investigate the physiological and biomechanical determinants of sprint ability following variable intensity exercise when roller ski skating. As expected, the physiological and biomechanical responses fluctuated in response to the changes in terrain during the simulated 21-min mass-start. Directly following this approach, the performance of skiers was ranked by TTE on the AOS. Here, better performance rank was associated with higher VO_2*Max*_ and GE as well as the ability to ski on a lower relative intensity (i.e., %HR_*Max*_, %VO_2*Max*_, and RPE) during the initial 21-min of the simulated mass-start. In addition, the potential to increase HR and VO_2_ in the AOS, beyond the corresponding values achieved during the initial 21-min of the simulated mass-start, correlated with performance. Finally, greater utilization of the G3 sub-technique in the steepest uphill, as well as a trend for longer CL during the AOS, were associated with better performance.

In this first study exploring physiological and biomechanical performance-determining variables in an experiment aiming to simulate the mass-start event in XC skiing, we observed increased VO_2_ and HR values from the uphills and reduced values from the downhills and flat terrain during the initial 21-min, as previously described in time trials and during high-intensity training in varying terrain (Bolger et al., 2015; Solli et al., 2018; Haugnes et al., 2019a). However, both HR and VO_2_ had a delay in the response to the changing workload as also shown previously (Gløersen et al., 2018, 2020), which is probably due to the combination of a real delayed physiological response (Barstow and Molé, 1991) in addition to measurement delays (e.g., low resolution of VO_2_ data and a delay due to data processing and averaging in the HR monitor). While the mean lap values for HR had a significant lap-to-lap drift, the mean VO_2_-values for each lap remained stable throughout the entire 21-min. However, the large individual differences in the relative intensity (i.e., %VO_2*Max*_ and %HR_*Max*_), and thereby in the potential to increase HR and VO_2_ in the AOS beyond the values achieved during the 21-min protocol, indicate that some skiers were working close to their maximum in the initial part of the mass-start. Accordingly, some skiers required one or more breaks, whereas others could perform the entire protocol quite comfortably.

In general, a similar picture is mirrored by the TSI values both for arms and legs, although the mean values for the different segments were not significantly different. The TSI_*Arm*_ and the distribution of power between poling and ski push offs had a significant lap-to-lap drift, with higher oxygenation saturation and more upper body effort in the first lap compared to the last lap. The shift in power distribution could have been done intentionally to save the legs toward the end, or possibly because the skiers got more fatigued in the upper than the lower body (i.e., reduced TSI_*Arm*_) and therefore generated relatively more power from the legs. The latter finding is in line with two previous studies were elite skiers performed diagonal stride (Björklund et al., 2010) and double poling (Stöggl et al., 2013). These studies showed that O_2_ extraction was lower and blood lactate production higher in the arms than the legs. This could indicate more fatigue in the arms relative to the legs and might be the reason why the skiers in our study used more relative power from the legs toward the end of the 21-min bout. In addition, this could also explain why the mean value for TSI_*Arm*_ was lower than for TSI_*Leg*,_ and why TSI_*Arm*_ had a small, but significant lap-to-lap drift. However, this lap-to-lap drift in power distribution did not influence CL and CR, both varied according to incline, speed and sub-technique utilization, but showed the same pattern within each sub-technique for all laps (i.e., no drift over time occurred).

The ability to perform well during the AOS at the end of the variable exercise during the simulated mass-start was largely correlated with both GE and VO_2*Max*_ (Figure 6 and Table 4), which allowed better skiers to work on a lower relative intensity during the initial part of the simulated mass start. Accordingly, a large correlation between performance and measures of intensity during the simulated mass-start (%HR_*Max*_, %VO_2*Max*_, and RPE) was present (Figure 5 and Table 4). This implies that a combination of higher aerobic energy delivery capacity and better efficiency allows the best performing skiers to start the AOS with less accumulation of fatigue and/or more anaerobic energy left. Both VO_2*Max*_ and GE have been shown to differentiate skiers on different performance levels and to allow skiers to utilize a higher aerobic power during time-trial competitions (Sandbakk and Holmberg, 2017). However, the novelty of this study is that these capacities seem to play a different role in mass-starts than shown for time-trials. Instead of using a superior VO_2*Max*_ and GE to increase speed, which is normally the case during time-trials, our findings imply that skiers who score high on these performance-determining variables can save energy and are therefore able to utilize their “reserves” better at the end of the race.

The best skiers were less fatigued after the 21-min initial part with variable intensity exercise with set speeds (i.e., simulating the conditions achieved in a mass-start race) and showed a better ability to increase VO_2_ and HR with gradually increasing speed during the AOS. In contrast, the lower-performing skiers were not able to reach VO_2_-values above those achieved during the steepest uphill in the main part of the mass-start, which may explain their limited ability to reach high speeds during the AOS. Specifically, the skiers ranked 6–13 in this study reached similar or higher HR and/or VO_2_ values during the initial 21-min compared to the AOS, while the HR and VO_2_ values for the top ranked skiers were (much) higher during AOS. Although micro-pacing in XC skiing includes higher effort uphill and downregulation of effort in downhills (Gløersen et al., 2018; Karlsson et al., 2018; Stöggl et al., 2018b), it seems important for skiers to work below a certain threshold also in the steepest uphills, allowing them to recover sufficiently in the subsequent downhills. This is for example shown in a 15-km simulated time-trial race (Gløersen et al., 2020), where elite skiers repeatedly attained substantial oxygen deficits in uphill segments. However, the deficits for each segment in that study were relatively small compared to their maximal accumulated oxygen deficit (MAOD), and within a level that could rapidly be recovered. Still, the total accumulated race O_2_ deficit was several times the MAOD, suggesting that this is an important energy contribution for an optimally paced race. Gløersen et al. (2020) argued that the ability to repeatedly use and recover the energy is an important performance indicator. In addition, a previous study comparing elite and lower-level skiers alternating between 3 min at 90% and 6 min at 70% of VO_2*Max*_ (Bjorklund et al., 2011) showed that lower-level skiers were less able to reduce BLa during the 70% intervals compared to elite skiers, even though there was no significant difference in BLa between the two groups after the first 90% interval (Bjorklund et al., 2011). These findings support the results presented here, illustrated by how the positive pacing strategies (involuntary) applied by the lower performing skiers in the initial 21-min limits their ability to recover and reach their full potential when sprinting at the end of the protocol.

In individual time-trial competitions, better-performing skiers utilize a more even pacing strategy than their lower-performing peers, who use a distinct positive pacing strategy and accumulate fatigue to a greater degree early in the race (Losnegard et al., 2016; Stöggl et al., 2018a, 2020). Our present results show that the forced pacing applied by the lower performing skiers in the simulated mass-start, which was too positive, forced them to accumulate fatigue in the initial part of the mass-start, which might have limited their ability to reach their full aerobic potential in the AOS. This novel finding provides important information about the effect of pacing on energetic capacity, with relevance both to mass-start events in XC skiing and other endurance sports.

Sub-technique selection in the steepest uphill, which had the highest workload, resulted in a very large Spearman’s correlation with performance, where the best skiers used more G3 at the expense of G2. This finding is in line with conclusions from two recent reviews (Stöggl et al., 2018a; Zoppirolli et al., 2020), where performance was linked to the ability to maintain speed in a specific section of a race. In our study, the skiers used the same speed in all similar terrain sections, but in line with the differences in relative intensity during the mass-start, also sub-technique selection was clearly differentiating performance levels. Specifically, the sub-technique selection in the steepest uphill divided the skiers in two groups, were only the best skiers utilized G3. In contrast, the 3 skiers who only used G2 in this section were in the group of lower-performing skiers requiring one or more breaks. This is further exemplified when correlating the 9 skiers performing the entire protocol using Pearson’s correlation, in which the significance between sub-technique selection and performance disappeared.

Coinciding with less tiredness and better aerobic power during the AOS, better performing skiers also showed the ability to concurrently produce longer CL and thereby have a lower CR at the set speed than their lower-performing peers. Two recent reviews define CL to be a trustable significant performance indicator, while CR is to a lower degree associated with performance (Stöggl et al., 2018a; Zoppirolli et al., 2020). However, none of the previous studies have examined temporal patterns in a finish-sprint where skiers had various degrees of accumulated fatigue as often occurring during a mass-start race. It should, however, be noted that these correlations were not significant when correlating the 9 skiers performing the entire protocol.

Relative power distribution between poles and skies, and between the power from left and right pole, displayed large variation between skiers for all sub-techniques, but we found only small, non-significant correlations with performance. This is in contrast to the conclusions from the meta-analysis by Zoppirolli et al. (2020), where more equal power distribution between sides was related to better performance. However, the large differences revealed in the use of sides and in the distribution of power from skis and poles may still be important information for each skier and can inform further technical development in training and competition. The same applies for oxygen saturation level in the muscles of arms and legs, where large individual differences occurred and only small to moderate correlations were found.

### Strength and Limitations

The present study was performed indoors while roller ski skating on a treadmill, where both physiological and biomechanical variables can be measured more correctly and detailed than during a real mass-start race performed outdoors on snow. This approach induced both strengths and limitations, with the ecological validity being particularly limited compared to studies on snow where interactions between skiers, tactics and drafting would play main roles. Accordingly, this study aimed to examine specific components with high relevance for the mass-start race, such as the cost of skiing a given track with a set workload and the subsequent effect on the ability to sprint at the end of the race. In this context, our protocol excludes the variable draft from skiing in a group and other aspects related to group dynamics. While this aspect limits the ecological validity, our protocol assures that all skiers were performing at the same prescribed speed and incline, allowing us to study the underlying physiological and biomechanical mechanisms and generalize more on the impact of these variables on performance. Also, the inclusion of a 21-min low-intensity familiarization session before the mass-start strengthen our study by securing that all skiers were fully warmed up and familiarized with the equipment, the specific treadmill and inclines, as well as the track profile.

## Conclusion

In this first study focusing specifically on performance related to the mass-start event in XC skiing by designing a protocol with variable intensity exercise with preset speeds and inclines followed by an AOS, the physiological and biomechanical variables fluctuated according to the changes in the simulated terrain, with a significant time-delay between a change in terrain and the physiological response and a lap-to-lap drift in %HR_*Max*_, TSI_*Arm*_ and power distribution between poling and ski push-offs. VO_2*Max*_ and skiing efficiency were significant performance-determining variables for simulated mass-start performance, enabling lower relative intensity during the initial phase, which likely caused less accumulation of fatigue when entering the final AOS. Subsequently, better performance was associated with more utilization of the demanding G3 sub-technique in the steepest uphill, and physiological reserves allowing better-performing skiers to utilize a larger portion of their aerobic potential and achieve longer CLs and higher speed during the AOS. Overall, our approach provides novel understanding of important mechanisms relevant for the mass-start events and provides a good starting point both for digging deeper into these mechanisms and opens for more applied approaches performed outdoors in future studies.

## Data Availability Statement

The data are not publicly available due to privacy concerns. Requests for accessing the dataset should be directed to TS, trine.seeberg@sintef.no.

## Ethics Statement

Ethical review and approval was not required for the study on human participants in accordance with the local legislation and institutional requirements. The patients/participants provided their written informed consent to participate in this study.

## Author Contributions

All authors contributed to the overall concepts, protocol, sensor setup and framework presented in the manuscript, and to the revision and approval of the submitted version. DN, JD, KS, and PH conducted the data collection. TS, ØS, JK, and JT prepared the manuscript. DN calculated GE. JD calculated power distribution. JK synchronized the sensor data. JT contributed with framework for sub-technique classification. ØS provided expert knowledge of the field and was responsible for designing the experiment. Finally, TS developed model for cycle detection and sub-technique classification, explored and analyzed data, made figures and tables, and was responsible for preparing the manuscript.

## Conflict of Interest

The authors declare that the research was conducted in the absence of any commercial or financial relationships that could be construed as a potential conflict of interest.
